# Regulation of BBB function and pathological evolution of PD by microenvironment “spatiotemporal gradient”: unique advantages of microfluidic chips

**DOI:** 10.3389/fnagi.2025.1599509

**Published:** 2025-07-09

**Authors:** Sixun Yu, Lingli Jiang, Min Song, Tao Yang, Ma Yuan, Xin Chen, Haifeng Shu

**Affiliations:** Department of Neurosurgery, Western Theater General Hospital, Chengdu, China

**Keywords:** microfluidic chip, blood-brain barrier, Parkinson’s disease, spatiotemporal gradients, organoid models, pathological simulation

## Abstract

Parkinson’s disease (PD), a prevalent neurodegenerative disorder, exhibits an exceedingly intricate pathological process characterized by multifaceted neuronal loss, inflammatory responses, protein misfolding, and blood-brain barrier (BBB) dysfunction. In the pathogenesis of PD, the BBB serves not only as a protective interface for the central nervous system but also actively contributes to the regulation of neural microenvironment homeostasis. Consequently, its impaired functionality can markedly exacerbate disease progression. Within the *in vivo* microenvironment, factors such as chemical gradients, fluid shear stress, and physical-mechanical signals play pivotal roles in modulating cellular behavior and organ function. The spatiotemporal dynamics of these gradients critically influence BBB integrity and neuroinflammatory responses. However, traditional *in vitro* models struggle to faithfully replicate such multidimensional dynamic microenvironmental changes. Recently, microfluidic chip technology has emerged as a transformative platform capable of simulating *in vivo* conditions through precise control of microenvironmental spatiotemporal gradients. This review examines the advancements of microfluidic chips in reproducing *in vivo* dynamic microenvironment gradients, regulating BBB function, and elucidating the pathological evolution of PD. It delves into the fundamental principles of microfluidic technology, gradient generation and control methodologies, and provides examples of BBB organoid models and PD pathological environment simulations constructed on this platform. Additionally, it systematically evaluates the technical bottlenecks, standardization challenges, and data integration issues associated with current model development, while exploring the potential for future technological convergence and interdisciplinary collaboration in advancing PD precision simulation and personalized treatment.

## 1 Introduction

Parkinson’s disease (PD) is the second most prevalent neurodegenerative disorder, affecting over 10 million individuals globally. With the aging of the global population, the total number of PD cases is projected to increase steadily (Tysnes and Storstein, 2017). Clinically, PD is defined by the progressive degeneration of dopaminergic neurons in the substantia nigra pars compacta, resulting in hallmark motor symptoms such as resting tremor, bradykinesia, and rigidity, as well as a variety of nonmotor features, including cognitive impairment and autonomic dysfunction (Coundouris et al., 2020). Additionally, PD demonstrates significant heterogeneity in symptom manifestation, age at onset, progression rate, and individualized treatment responses. Although therapies such as levodopa and deep brain stimulation can alleviate symptoms, they are unable to prevent or reverse neuronal loss. Therefore, elucidating the multifactorial pathogenesis of PD is an urgent priority (Chakraborty et al., 2020; Han and Hu, 2020; Reich et al., 2022). Emerging evidence indicates that the BBB serves not merely as a passive barrier but also as an active and dynamic regulator. Dysfunction of the BBB actively contributes to the progression of PD via complex spatiotemporal interactions among neuroinflammation, α-synuclein (α-syn) propagation, and oxidative stress (Pan and Nicolazzo, 2018; Lazdon et al., 2020; Tofaris, 2022).

In recent years, the role of the BBB in PD has evolved from a “static barrier” to a “dynamic regulator.” For instance, clinical evidence demonstrates that the expression levels of claudin-5 and occludin in cerebral microvessels of PD patients are significantly downregulated, and the function of the efflux transporter P-glycoprotein (P-gp) is impaired, leading to increased BBB permeability (Lee and Pienaar, 2014; Al-Bachari et al., 2020). This disruption facilitates the infiltration of peripheral inflammatory factors (e.g., IL-6, TNF-α) and neurotoxins (e.g., 6-hydroxydopamine, 6-OHDA) into the brain parenchyma via a compromised BBB, establishing local concentration gradients. These gradients further activate microglia and promote the pathological aggregation and propagation of α-syn (Pediaditakis et al., 2021). In addition, concentration gradients of TNF-α can activate the NF-κB signaling pathway in endothelial cells, subsequently resulting in the degradation of tight junction proteins and thus perpetuating a vicious cycle of neurovascular uncoupling (Nallasamy et al., 2021). Metabolic disturbances, including mitochondrial dysfunction and glucose hypometabolism, generate a spatial gradient in energy distribution, which further exacerbates oxidative damage within vulnerable neuronal populations (Zheng et al., 2021). These findings highlight the pivotal role of the BBB in PD pathology. Nevertheless, traditional studies predominantly focus on single functional dimensions of the BBB (e.g., permeability assessments), while overlooking the critical property that BBB function is finely regulated by spatiotemporal dynamic changes within the microenvironment.

The *in vivo* microenvironment forms a complex four-dimensional dynamic network that integrates chemical gradients, biomechanical signals, and cell–cell interactions, which is defined by its “spatiotemporal gradient” characteristics. The spatiotemporal gradient within the *in vivo* microenvironment refers to the synergistic effects of chemical factors (e.g., oxygen, inflammatory mediators, neurotrophic factors) and physical factors (e.g., shear stress) on both spatial distribution and temporal dynamics. For instance, neurotrophic factor concentration gradients in the brain parenchyma direct neural stem cell migration, while vascular endothelial cells preserve barrier polarity under the influence of blood flow-induced shear forces (Xiao et al., 2018; Pediaditakis et al., 2021; Tunç et al., 2021). These spatiotemporally dynamic signals are indispensable for the development of the BBB, the maintenance of homeostasis, and the repair of pathological damage. Nevertheless, conventional *in vitro* models, such as the Transwell system, are constrained by static culture conditions. Such models fail to replicate the physiological shear forces experienced by endothelial cells or establish chemical gradients with precise spatiotemporal resolution, thereby creating a substantial discrepancy between the model and the authentic *in vivo* microenvironment (Petrovskaya et al., 2022).

**Figure 1:**
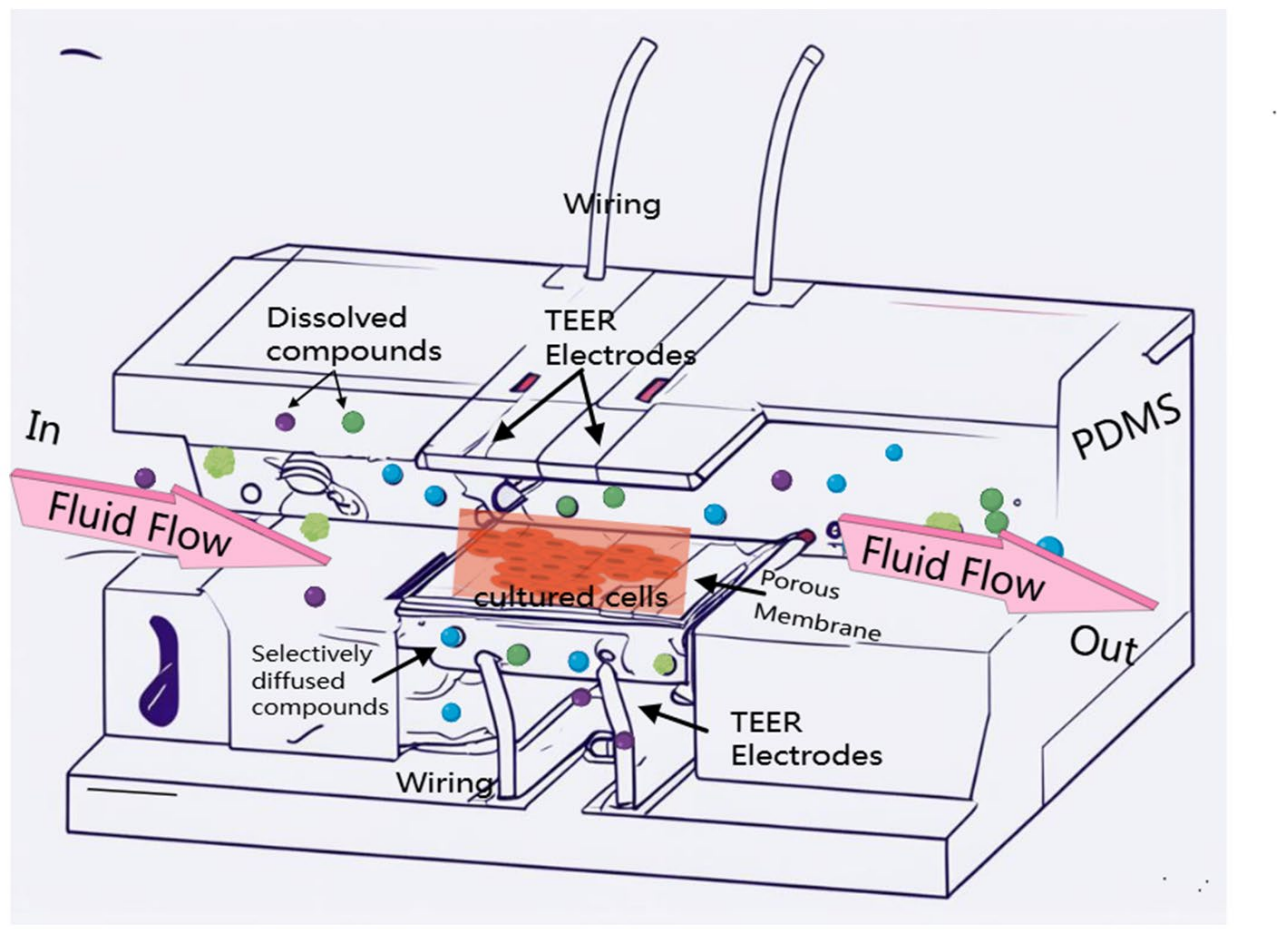
BBB microfluidic chip: Herein, we display an advanced multilayer microfluidic device inspired by the design of Ross Booth et al., which comprises four PDMS baseplates, two glass layers, and a centrally positioned porous polycarbonate film within the PDMS structure. The integrated device features two vertically intersecting channels designed for dynamic fluid introduction, a porous membrane located at the channel intersection to facilitate cell culture, and embedded electrodes for monitoring transepithelial electrical resistance (TEER).

The emergence of microfluidic chip technology introduces a transformative paradigm for overcoming this bottleneck. As depicted in Figure 1, a design scheme leveraging BBB microfluidics technology explicitly highlights its technical characteristics. Through the integration of a microchannel network with a fluid control system in a co-design framework, this technology is capable of precisely replicating the *in vivo* microscale dynamic environment, encompassing chemokine diffusion, intermittent shear stress gradients, and the spatial organization of multi-cellular structures. Further advantages of this technology will be progressively unveiled in subsequent sections (Kimura et al., 2018; Jørgensen et al., 2020). The BBB-on-chip model developed based on this technology not only enables the bioprinting of 3D blood vessel structures but also dynamically simulates the interactions of neurovascular units during the pathological progression of PD by incorporating neurons, astrocytes, and microglia (van der Helm et al., 2016; Kawakita et al., 2022). More significantly, the microfluidic platform facilitates real-time monitoring of barrier permeability, intercellular communication, and the spatiotemporal evolution of pathological protein aggregation, offering a unique perspective to investigate the cascade of “BBB dysregulation, neuroinflammation, and protein misfolding” in PD progression (Vatine et al., 2019). Additionally, it creates opportunities for high-throughput drug screening and personalized medicine strategies.

This review highlights the pivotal role of microfluidic chip technology in elucidating the relationship between the spatiotemporal dynamic microenvironment of the BBB and the pathology of PD. It systematically investigates the molecular mechanisms underlying the dynamic gradient (chemical, physical, biomechanical) regulation of BBB dysfunction and the pathological cascade of PD. Notably, this review does not delve into or discuss the technical details of microchip fabrication, which have been extensively summarized in prior studies. Our objective is to construct a theoretical framework linking the temporal and spatial gradients of the microenvironment with the progression of neurodegenerative diseases by integrating state-of-the-art research findings. Furthermore, we propose potential development directions to offer new insights into dynamic regulation strategies targeting the BBB.

## 2 Microfluidic chip technology and the principle of spatiotemporal gradient

### 2.1 Basic structure and working principle of microfluidic chip

In recent years, microfluidic technology has experienced rapid advancements. At its core, this technology employs micron-scale channels to precisely manipulate fluids, thereby creating an *in vitro* microenvironment that closely mimics biological conditions within living organisms. Microfluidic chips are typically fabricated from transparent, highly elastic, and biocompatible materials such as polydimethylsiloxane (PDMS), polymethyl methacrylate (PMMA), glass, and silicon-based substrates. These materials not only facilitate ease of processing and functionalization but also ensure the biosafety necessary for cell culture (Chung et al., 2010; Wu et al., 2020; Kawakita et al., 2022). The fundamental design architecture of these chips generally comprises key components including microflow channels, infusion ports, sampling outlets, microreaction chambers, and gradient generation zones, all interconnected via meticulously engineered geometric structures. Common design strategies encompass sandwich configurations, parallel layouts, and three-position tubular structures (Nakagawa et al., 2009; Tóth et al., 2011; Modarres et al., 2018). Such designs collectively form an integrated system capable of simulating the motion and transport processes of complex fluids within biological systems.

In microfluidic chips, the fundamental principles of fluid mechanics are predominantly governed by laminar flow characteristics and low Reynolds number regimes, enabling smooth and predictable fluid movement within microchannels without turbulence. Hagen–Poiseuille’s law provides an accurate description of the relationship between pressure differences, flow rates, and fluid viscosity in these microchannels, facilitating precise flow control (Griep et al., 2013; Amirifar et al., 2022). Furthermore, by designing specific channel networks (e.g., gradient generators, forked networks, or tree-like structures) and incorporating micromixer technologies, stable and controllable chemical and physical gradients can be established within the chip. For instance, introducing two solutions with differing concentrations at the channel entrance and leveraging the diffusion properties of microchannels enables the generation of precise spatial gradient distributions. Additionally, the shear forces exerted by fluids within the channels can be finely modulated, which is crucial for replicating the mechanical stimuli experienced by cells under physiological conditions (Chung et al., 2010; Mehta et al., 2022). This temporal and spatial gradient, generated based on fluid mechanics and diffusion principles, can faithfully replicate the dynamic microenvironment within the body. Consequently, it provides a robust experimental platform for investigating the function of the BBB and complex neurological diseases such as PD. In general, the fundamental architecture of microfluidic chips hinges on highly integrated microstructure design and precisely controllable fluid dynamics. By selecting appropriate materials and fabrication processes, these chips not only offer a transparent, stable, and biocompatible experimental platform but also generate dynamic gradients to meet specific requirements by modulating channel geometry and fluid parameters. These features enable microfluidic chips to exhibit unique technical advantages in reconstructing physiological temporal and spatial gradients, simulating *in vivo* environments, and studying disease progression, thereby offering a novel approach to overcome the limitations of traditional static *in vitro* models.

### 2.2 Concept analysis of “spatiotemporal gradient”

In living organisms, the microenvironment in which cells reside is not static but rather a dynamic system that continuously evolves with spatial location and temporal progression. The term “spatiotemporal gradient” denotes the integrated effect of both the spatial and temporal gradients present within this microenvironment. The spatial gradient primarily reflects variations in the concentration or distribution of chemical factors, nutrients, and oxygen across different locations. In contrast, the temporal gradient refers to the dynamic fluctuations of these concentrations or states over time, encompassing both short-term pulse-like changes and continuous flow dynamics. Together, these two gradients are intricately intertwined, shaping the external signals that cells sense and respond to, thereby profoundly influencing cell fate, signal transduction, and organ function (Doupé and Perrimon, 2016; Economou and Hill, 2020).

In terms of spatial gradients, the chemical concentration gradient prevalent in biological systems serves as a typical example. During development, morphogen distribution frequently exhibits a continuous transition from high to low concentrations, providing critical “positional” information that guides cell differentiation and organ formation (Huang and Saunders, 2020; Hubatsch and Goehring, 2020). Similarly, in the establishment of the BBB, the non-uniform distribution of inflammatory factors, cytokines, nutrients, and oxygen within the local microenvironment is regarded as a key factor influencing cellular interactions and the formation of tight junctions (Oghabian et al., 2022). Moreover, the spatial chemical gradients generated by mixing solutions of varying concentrations at multiple inlets in microfluidic platforms offer an essential approach for simulating these heterogeneous conditions observed *in vivo*.

The temporal gradient pertains to the variation of signals across time scales. Under natural conditions, environmental signals are typically not static but exhibit periodic, impulsive, or continuous changes. For instance, the supply of oxygen and nutrients in the blood exhibits inherent fluctuations (Smith and Ainslie, 2017); cytokine levels also undergo significant oscillations within short timeframes during inflammatory processes (Liu et al., 2021). These dynamic variations play a crucial role in modulating cell function, inducing stress responses, and regulating gene expression. However, traditional static culture models often fail to capture such temporal dynamics, making it challenging to fully reconstruct the intricate *in vivo* signaling networks (Zhu et al., 2023).

The common types of gradients are not only chemical concentration gradients but also encompass several other aspects: (1) Fluid shear gradients: In vascular systems and microfluidic chips, variations in fluid shear stress can directly influence cell morphology, the tight junctions of endothelial cells, and gene expression, thereby modulating vascular homeostasis and functionality (Galie et al., 2014; Dessalles et al., 2021). (2) Temperature gradients: The heterogeneity of local temperature distribution plays a critical role in biological processes such as tissue repair, inflammatory responses, and tumor microenvironment regulation. For instance, Kumar et al. (2023) employed fluorescent polymer nanothermometers (FPNTs) to measure intratumoral temperature distributions in co-cultured three-dimensional tumor spheroids. (3) Mechanical stress gradient: Spatial and temporal variations in mechanical properties, such as matrix stiffness and pressure distribution, play a critical role in cellular responses. These mechanical cues are transmitted via the cytoskeleton to modulate cell proliferation, migration, and differentiation. For instance, stem cell phenotypes can be influenced by mechanical factors, including matrix stiffness and surface topography. Mesenchymal stem cells cultured on a soft matrix, which mimics the elasticity of brain tissue, differentiate into neuronal precursors. A matrix with intermediate stiffness, similar to that of muscle tissue, promotes myogenic differentiation. Rigid substrates that replicate the stiffness of collagen-rich bone tissue induce osteogenic differentiation (Yang et al., 2015).

In summary, the spatiotemporal gradient is not merely a simple superposition of individual gradients but rather a sophisticated system characterized by mutual coupling and feedback regulation. *In vivo*, this mechanism of spatiotemporal gradient regulation enables all cell types to respond precisely when adapting to external stimuli, which is essential for maintaining organ function and coordinating interactions within multicellular systems. In recent years, aided by microfluidic chip technology, researchers have progressively achieved *in vitro* reconstruction of such complex gradients, significantly advancing biomedical research toward higher levels of dynamic simulation and precise regulation (Bonifácio et al., 2019).

### 2.3 Examples of application of microfluidic platform in physiological and pathological simulation

The core advantage of microfluidic technology resides in its capacity to precisely simulate dynamic spatiotemporal gradients *in vivo*, demonstrating broad applicability in physiological and pathological research. When integrated with key studies in neuroscience, cardiovascular systems, and oncology, the following elucidates how this technology addresses critical scientific questions via the regulation of spatiotemporal gradients.

In neuroscience research, microfluidic platforms serve as a transformative tool for analyzing BBB function. By incorporating the multicellular components of the neurovascular unit (NVU) into bionic chips, studies have demonstrated that fluid shear stress gradients play a regulatory role in maintaining BBB integrity. For instance, endothelial cells cultured in MOTF biochips exhibit enhanced BBB shielding function under high shear stress conditions. This improvement is associated with the upregulation of PECAM-1 and ZO-1, key regulators of endothelial tight junction integrity (Crockett et al., 2010; Cong and Kong, 2020). Additionally, prolonged lateral contact between thickened endothelial monolayer cells, mediated by cytoskeletal rearrangement amplification, further enhances the barrier function of the endothelial layer under high shear stress stimulation (Cong and Kong, 2020). The constructed neurotoxicity gradient model revealed that an acrylamide concentration gradient could precisely modulate the axonal degeneration rate of hippocampal neurons and induce morphological changes in astrocytic foot processes. Additionally, ZO-1 protein expression exhibited a 50% gradient decline corresponding to the acrylamide concentration gradient, visually replicating the pathological process of neuronal injury caused by the leakage of neurotoxic substances across the barrier (Li et al., 2012).

Similarly, the universality of the microfluidic platform has been validated in cross-disciplinary research. In cardiovascular studies, researchers simulated a vascular microenvironment with pulsatile shear force gradients (1–10 dyn/cm^2^) and demonstrated that dynamic mechanical stimulation induces endothelial cell alignment while significantly upregulating the expression of VEGFR2, a key marker of angiogenesis. This finding underscores the critical role of physiological gradients in maintaining vascular homeostasis (Christoffersson et al., 2018). In oncology, drug screening was performed using a concentration gradient chip (Poddar et al., 2024; Aydin et al., 2024). Song et al. (2023) developed a novel bionic three-dimensional tumor culture and clinical drug screening platform for primary pancreatic cancer cells by leveraging microfluidic electrospray technology. By integrating encapsulated tumor spheroids into a microfluidic chip equipped with a concentration gradient channel and culture chamber, high-throughput evaluation of chemotherapy regimens under varying concentrations can be achieved, providing essential parameters for optimizing drug delivery systems. These multiscale investigations reveal that microfluidic technology not only overcomes the limitations of traditional static culture models through precise spatiotemporal gradient control but also offers a systematic approach to studying multi-factor coupled pathological mechanisms.

Based on these application examples, the microfluidic platform has emerged as a critical tool for physiological and pathological studies due to its ability to replicate *in vivo* temporal and spatial gradients. Subsequently, we will systematically investigate how chemical and physical gradients dynamically influence the integrity of the BBB via molecular mechanisms, such as endothelial cell phenotypic remodeling and regulation of tight junction protein expression. Furthermore, we will explore how these processes contribute to the pathological progression of Parkinson’s disease.

## 3 Regulation of BBB function by microenvironmental gradients

### 3.1 Introduction to BBB structure and multi-level functions

The BBB serves as a critical protective barrier for the central nervous system. Its primary role is to maintain the homeostasis of the brain’s microenvironment, prevent harmful substances from infiltrating nervous tissue, and provide essential nutritional support and signal transmission for neurons (Whelan et al., 2021). Recent studies have revealed that the BBB is not merely a single-layered structure but rather a complex “neurovascular unit” comprising endothelial cells, pericytes, astrocytes, and other cell types. These cells interact intricately to jointly sustain the multi-level functionality of the BBB (Kadry et al., 2020). As the principal constituent cells of the BBB, cerebral microvascular endothelial cells exhibit a highly specialized phenotype. The tight junctions between these cells are formed by proteins such as claudin-5, occludin, and zonula occludens-1 (ZO-1). These proteins are tightly arranged, significantly restricting the passage of substances through the intercellular space and thereby forming an effective physical barrier (Medina-Flores et al., 2020). Pericytes, which are located around the microvascular endothelial cells and embedded within the basal membrane, regulate endothelial cell growth, differentiation, and the maintenance of tight junctions via both direct cell–cell contact and the secretion of various growth factors and cytokines (Roth et al., 2024). Recent *in vivo* imaging and functional studies have conclusively demonstrated that perivascular cells play an indispensable role in maintaining microvascular stability and regulating local blood flow dynamics. Their dysfunction or loss is strongly associated with increased BBB permeability and localized inflammatory responses (Nicoli and Grutzendler, 2021). Astrocytes, by enveloping the outer side of blood vessels with their abundant peripheral structures (foot processes), provide structural and functional support to endothelial cells. They supply essential metabolites and growth factors, such as VEGF, and secrete a variety of cytokines, including matrix metalloproteinases and inflammatory mediators, which collectively regulate the physiological state of endothelial cells (Heithoff et al., 2021). In recent years, research employing *in vivo* imaging and molecular marker technologies has revealed that astrocytes actively participate in responding to oxidative stress, repairing vascular damage, and modulating neuroinflammation, thereby playing a pivotal role in BBB remodeling and disease defense mechanisms (Heithoff et al., 2021; Schiera et al., 2024).

The integrity of the BBB relies on the dense cell layer formed by endothelial cells and its molecular mosaic of tight junctions. Key signaling pathways, such as Wnt/β-catenin and Notch, regulate gene expression and cell polarity associated with these tight junctions, thereby ensuring the physical robustness of the barrier (Von Stetina et al., 2018; Song et al., 2022). Simultaneously, the BBB exhibits highly selective transport functions, including receptor-mediated transport, carrier-mediated transport, and transcytosis, which collectively control the flux of nutrients, neurotransmitters, hormones, drugs, and other molecules (Saidijam et al., 2018). For instance, efflux transporters such as P-glycoprotein (P-gp) and breast cancer resistance protein (BCRP) act as “gatekeepers” to restrict the entry of xenobiotics into the brain, and their activity directly influences drug distribution within the central nervous system (Saidijam et al., 2018). Moreover, deep bidirectional signal communication is established among the constituent cells of the BBB via extracellular vesicles, cell adhesion molecules, and gap junctions. This transcellular communication network enables the neurovascular unit to sense local environmental changes in real time, such as inflammatory stimuli, metabolic abnormalities, or mechanical stress, and rapidly adjust endothelial tight junctions and transport mechanisms to maintain overall barrier stability (Von Stetina et al., 2018). Recent single-cell transcriptomic studies have further elucidated the complex and dynamic interactions between endothelial cells, pericytes, and astrocytes, underscoring the pivotal role of intercellular communication in preserving BBB homeostasis and responding to neuropathological conditions (Giannoni et al., 2018). The composition and key aspects of the BBB are illustrated in Figure 1.

### 3.2 Influence of chemical and physical gradients on endothelial cell phenotypes and tight junctions

**Figure 2:**
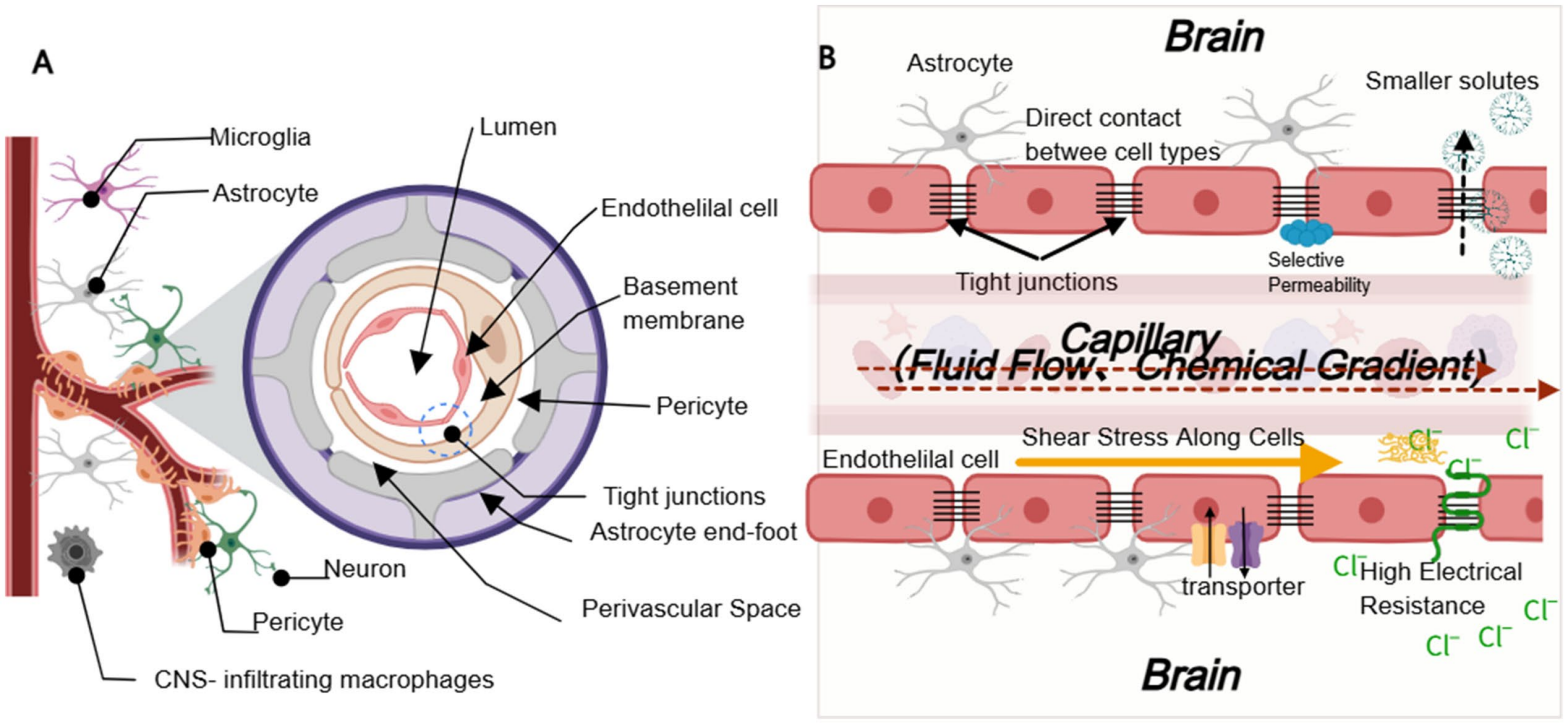
The Composition and Key Features of the BBB **(A)** Cross-sectional view illustrating the neurovascular units comprising endothelial cells, pericytes, astrocytes, basement membranes, neurons, and immune cells. **(B)** An effective in vitro BBB model should successfully incorporate the following essential characteristics: (1) endothelial cells expressing functional tight junctions; (2) co-culture with astrocytes to mimic physiological conditions; (3) exposure to shear stress to simulate vascular dynamics; (4) selective permeability to compounds; and (5) high trans-endothelial electrical resistance (TEER) at tight junctions.

As a critical protective barrier of the central nervous system, BBB endothelial cells establish a physical barrier through the formation of tight intercellular junctions (primarily comprising proteins such as claudin-5, occludin, and ZO-1). In recent years, numerous studies have demonstrated that both chemical gradients (such as oxygen, nutrients, and cytokines) and physical gradients (such as fluid shear stress and dynamic mechanical signals) within the endothelial microenvironment significantly regulate cell polarity, tight junction protein expression, and cellular function (see Figure 2).

#### 3.2.1 The regulatory role of chemical gradients in cellular processes

In different regions of the microvascular network, there are often significant oxygen distribution gradients. A hypoxic environment can lead to the up-regulation of hypoxia-inducible factor (HIF-1α) in endothelial cells, thereby activating downstream signaling pathways such as VEGF. This activation subsequently regulates the expression of tight junction proteins, including claudin-5 and occludin. For instance, a microfluidic model was employed to precisely construct oxygen gradient conditions. It was demonstrated that HIF-1α activation in hypoxic regions is closely associated with the remodeling of tight junction protein expression, providing a molecular basis for the formation of the BBB (Peña and Vazquez, 2020; Zheng et al., 2021). In addition, Juan et al. reported that insufficient oxygen and nutrient supply may upregulate factors such as vascular endothelial growth factor (VEGF) and epidermal growth factor (EGF), thereby promoting abnormal angiogenesis and compromising the structural integrity of the retinal blood barrier (BRB) (Peña and Vazquez, 2020). Secondly, the uptake of glucose, amino acids, and other essential nutrients by endothelial cells directly influences their metabolic state and cytoskeletal remodeling, which in turn indirectly regulates the stability of tight junctions. For instance, elevated levels of branched-chain amino acids (BCAAs) can induce inflammation and oxidative stress in endothelial cells, thereby promoting inflammatory cell adhesion and endothelial dysfunction (Zhenyukh et al., 2018). Moreover, cytokine gradients (e.g., TNF-α and IL-6) formed in the local inflammatory microenvironment can activate signaling pathways such as NF-κB, leading to the downregulation of tight junction protein expression and alterations in cell polarity (Voirin et al., 2020). Conversely, anti-inflammatory therapies are beneficial for reversing inflammation-induced barrier dysfunction (Matsuhisa et al., 2018). This process is particularly pronounced in acute inflammation and certain neurodegenerative diseases, highlighting the bidirectional regulation of endothelial barrier function by chemical gradients (Jeon et al., 2020).

#### 3.2.2 The regulatory role of physical gradients

The fluid shear force generated by blood flow has long been recognized as a “mechanical signal” for endothelial cells. Under stable shear stress, endothelial cells typically align along the flow direction, accompanied by cytoskeletal remodeling and redistribution of tight junction proteins, which enhances barrier function (Matsuhisa et al., 2018; Ryu et al., 2021). Moreover, dynamic mechanical signals, such as pulsatile or intermittent flow, can periodically activate the PI3K/Akt and MAPK signaling pathways, thereby regulating the local expression and reaggregation of proteins like occludin and claudin-5. Endothelial cells exhibit high sensitivity to changes in blood flow dynamics, and errors in mechano-transduction may lead to abnormal physiological functions of blood vessels, contributing to the onset and progression of various vascular diseases, including hypertension, thrombosis, aneurysms, and atherosclerosis (Ryu et al., 2021). By using microfluidic platforms to simulate variations in physical parameters, studies have demonstrated that different modes of shear force can induce immediate and reversible regulation of endothelial cell morphology and function, providing critical experimental evidence for understanding the role of physical signals in maintaining the BBB (Ryu et al., 2021).

In summary, endothelial cells exhibit remarkable sensitivity to both chemical gradients (e.g., oxygen, nutrients, cytokines) and physical gradients (e.g., fluid shear stress, dynamic mechanical stimulation). Through the activation of multiple signaling pathways, including HIF-1α, NF-κB, PI3K/Akt, and MAPK, these gradients synergistically regulate key processes such as the establishment of cell polarity, expression of tight junction proteins, and cytoskeletal remodeling. These processes constitute the fundamental molecular mechanisms underlying the maintenance of BBB physical barrier function.

In addition to the molecular regulation of individual endothelial cells, chemical and physical gradients collectively drive the entire process of BBB development, from its initial formation to a mature functional state, via cell–cell interactions and signaling exchanges within the neurovascular unit. This ensures the BBB’s dynamic adaptation to both internal and external environmental changes. *In vivo*, the BBB is not only composed of endothelial cells but also includes pericytes and astrocytes. Under the influence of various chemical gradients, these cells achieve cross-cellular signaling and cooperative regulation by secreting growth factors (such as VEGF and PDGF) and cytokines. Simultaneously, extracellular vesicles and gap junctions facilitate intercellular communication, enabling the entire BBB system to rapidly respond to fluctuations in local chemical and physical gradients.

## 4 The role of microenvironmental gradients in the pathological evolution of PD

### 4.1 Microenvironmental alterations during the pathological progression of PD

In recent years, an increasing number of studies have demonstrated that PD is not solely attributable to the selective loss of dopaminergic neurons but is also associated with multiple pathological processes, including oxidative stress, inflammatory responses, and metabolic disturbances within the local microenvironment. These pathological conditions often exhibit gradient distribution characteristics in the brain. Specifically, in affected regions, the concentrations of oxygen, nutrients, and cytokines display pronounced spatial or temporal imbalances, thereby creating a localized “gradient imbalance” state.

Oxidative stress is a critical factor in the degeneration of dopaminergic neurons in PD. Specifically, when the physiological REDOX balance in neurons is disrupted, it interferes with numerous biological processes, ultimately leading to apoptosis or necrosis. In PD patients, mitochondrial dysfunction and inadequate antioxidant defense mechanisms result in significantly elevated levels of reactive oxygen species (ROS), creating oxidative stress gradients around local nerve cells (Ryu et al., 2021). This state of localized hypoxia or high oxidative stress not only causes cellular damage but also induces protein modifications and misfolding, which subsequently promotes the abnormal aggregation of α-syn, serving as a key trigger for the pathological progression of PD. Consequently, many studies have focused on extracting antioxidant compounds from natural products as potential therapeutic strategies for PD (Ryu et al., 2021; Sharma et al., 2021).

Long-term low-grade chronic inflammation is a critical feature in the pathological progression of PD. Research has demonstrated that the local concentrations of pro-inflammatory cytokines, such as TNF-α, IL-1β, and IL-6, are markedly elevated in affected regions compared to adjacent normal areas, establishing a pronounced concentration gradient (Pajares et al., 2020; Forloni et al., 2021). This gradient-driven inflammatory milieu not only activates resident glial cells and peripheral immune cells but also disrupts the homeostasis of the neuro-microenvironment. Furthermore, it exacerbates the pathological aggregation of α-syn, thereby accelerating neurodegeneration (Pajares et al., 2020; Forloni et al., 2021). In addition, the energy metabolism in the brains of PD patients exhibits an imbalanced state. Multiple studies have demonstrated that mitochondrial dysfunction and glucose metabolism disorders result in gradient differences in energy supply and metabolite concentrations between diseased and healthy regions (Rocha et al., 2018). This metabolic gradient may potentiate local cellular stress responses and apoptotic signaling, thereby creating a conducive environment for the progression of neuronal degeneration and abnormal aggregation of α-syn.

In conclusion, oxidative stress, inflammatory response, and metabolic disorder in PD pathology are not isolated phenomena but dynamically interact to form a positive feedback loop through the spatiotemporal coupling of multiple gradients. Specifically, the local ROS gradient induces degradation of tight junction proteins in endothelial cells, thereby increasing BBB permeability. The extravasation of peripheral inflammatory factors, such as TNF-α, establishes a concentration gradient that activates microglia and facilitates the pathological propagation of α-syn. Simultaneously, the disruption of energy metabolism gradients exacerbates neuronal oxidative damage by inhibiting mitochondrial function. By simultaneously simulating these interacting gradients, microfluidic chips provide a powerful tool to unravel this intricate network.

## 5 Reproducing spatiotemporal gradients using microfluidic chips: experimental design and modeling construction

As an advanced platform capable of reproducing *in vivo* space-time gradients, microfluidic chips have gained widespread application in biomedical research over the past few years. Their success hinges on the precise control of multiparameter fluid dynamics and chemical gradients.

### 5.1 The strategy for chip design

The current chip design tends to integrate a multi-channel fluid control system and dynamic gradient generation technology. The design concepts are as follows: (1) Multi-channel configuration: parallel or cross channels are employed to generate simultaneous concentration gradients of varying levels, thereby fulfilling the simulation requirements for complex physiological microenvironments (Kawakita et al., 2022); (2) Dynamic gradient generation: by regulating flow rates through pumping control systems and micro-valves, precise adjustments to the amplitude and slope of gradients can be achieved across different time scales (Wang et al., 2021); (3) Online real-time monitoring: integrated micro-sensors, live cell imaging systems, or 3D printing technologies enable real-time monitoring of gradient distribution, fluid velocity, and changes in cellular behavior (Arandian et al., 2019; Meghani et al., 2020).

#### 5.1.1 Multi-parameter integrated control

Integrated control technology is the core of the platform. Current research has realized multi-parameter coordination of flow rate, gradient amplitude, and time regulation, and continuously optimized the material distribution in the chip through feedback control algorithm, thus truly simulating the complex space-time ladder in the body (Shinohara, 2016; Frossard et al., 2019). Therefore, in the past decade, the integration of inline sensors into BBB-on-a-chip devices has been an active research field, with TEER sensors being a typical example. These systems have been used in the study of neurodegenerative diseases such as Alzheimer’s disease (Mir et al., 2022; Palma-Florez et al., 2023).

### 5.2 Constructing functional BBB organoids or co-culture systems

The utilization of microfluidic chips for constructing functional BBB organoids or multicellular co-culture systems offers a distinctive platform to investigate the regulation of the neural microenvironment and the mechanisms underlying pathological processes.

#### 5.2.1 Construction of multi-cell co-culture system

The multi-cell co-culture of endothelial cells, pericytes, and astrocytes has been successfully established on the microfluidic chip platform, enabling an effective simulation of the neurovascular unit through precise control of gradient conditions. In this system, different cell types collaboratively maintain the structure and function of the blood-brain barrier (BBB) via intimate physical interactions and secretory signaling coupling (Cucullo et al., 2007; Booth and Kim, 2012; Canfield et al., 2017; Andrews et al., 2018). Multilevel co-culture not only ensures authentic cell–cell interactions but also provides a robust model for further investigation of cell behavior under gradient influences.

#### 5.2.2 Gradient regulation improves tissue maturity and functional stability

Using precisely controlled chemical and physical gradients within the chip, researchers can induce polarity remodeling in endothelial cells and upregulate tight-junction proteins, thereby enhancing the maturity and stability of the constructed BBB model (Booth et al., 2014; Shemesh et al., 2015). In the design of the micro-platform, the hydrogel’s performance and structural characteristics enable it to provide an appropriate spatiotemporal model for the biochemical and biomechanical processes that regulate cell behavior. Consequently, through dynamic gradient regulation, not only can the distribution of nutrients and signals under physiological conditions be accurately reproduced, but also the imbalances of oxygen, metabolites, and inflammatory factors under pathological conditions be effectively simulated. This provides an ideal experimental platform for studying neurodegenerative diseases, including BBB dysfunction in Parkinson’s disease (PD) (Canfield et al., 2017; Pasman et al., 2018; Dura et al., 2021).

## 6 Current status and prospects of a chip model that can simulate PD pathological environment

### 6.1 Feasibility of constructing PD microenvironment chip model

In recent years, a series of *in vitro* model experiments conducted using microfluidic and dynamic gradient simulation platforms have provided compelling evidence for investigating the relationship between gradient imbalances in the PD microenvironment and neurodegenerative changes.

#### 6.1.1 Dynamic gradients simulate the distribution of inflammatory factors

Kim et al. (2012) was the pioneer in constructing a microfluidic chip for studying WNT-β-catenin signaling and Rifes et al. (2020) subsequently utilized a microfluidic device to culture human embryonic stem cells and establish WNT gradient activation, thereby forming *in vitro* neural tissue. While this technology was primarily employed to investigate the differentiation and maturation processes of neural tissues, the dynamic gradient regulation approach implemented by the chip also served as a technical reference for subsequent simulations of inflammatory gradients in PD. By modulating the concentration distribution of inflammatory factors such as IL-6 and TNF-α within the chip, this model revealed the substantial impact of localized high-concentration areas on disrupting endothelial cell tight junctions and enhancing permeability. These findings suggest that variations in inflammatory gradients may contribute to the damage of peripheral nerve cells (Brown et al., 2014).

#### 6.1.2 Mitochondrial damage model under high glucose conditions

Some researchers have designed a single-channel microfluidic device capable of capturing *Caenorhabditis elegans* and enabling precise imaging of mitochondria within its body wall muscle. These microfluidic chips are employed to investigate the impact of hyperglycemia on mitochondrial fluorescence intensity, evaluate the extent of mitochondrial damage, and verify that methyl metformin mitigates the adverse effects of hyperglycemia on mitochondrial integrity (Sofela et al., 2020). This model elucidates how metabolic imbalance dynamically regulates glucose gradients and thereby exacerbates mitochondrial dysfunction in PD.

#### 6.1.3 Oxygen gradient and nerve cell damage model

Another study conducted by Taylor et al. utilized a microfluidics platform to construct a localized hypoglycemic and hypoxic model of neurons. While this model has primarily been applied in the investigation of cranial trauma, its characteristics closely resemble the oxygen supply deficiency environment induced by mitochondrial dysfunction in PD pathology. Therefore, it holds promise for adaptation to the study of hypoxic mitochondria in PD.

#### 6.1.4 “Substantia nigra on a chip” model and α-syn intercellular diffusion model

In a recent study by Pediaditakis et al. (2021), a “BBB chip” was utilized to co-culture BBB endothelial cells, pericytes, astrocytes, microglia, and dopaminergic neurons derived from human induced pluripotent stem cells. Within the brain channel of the chip, these cells were exposed to α*-syn* pre-formed fibrils, thereby inducing the pathogenesis of PD. This innovative “substantia nigra brain chip” not only recapitulates key pathological hallmarks of PD, such as the accumulation of phosphorylated serine 129 α-syn (pSer129-αSyn), reduced mitochondrial function, neuroinflammation, and neuronal loss, but also demonstrates disruption of the BBB, evidenced by increased permeability to various tracer molecules.

Fernandes et al. (2016) has developed a novel microfluidic cell culture platform for investigating communication between two distinct cell populations. The integration of microvalves in the device enables researchers to precisely control fluid pathways, the cellular microenvironment, and simulate paracrine signaling. Co-culture experiments were conducted to study the propagation of α-SYN between cells. Results indicated that α-Syn-GFP is not internalized by cells under normal conditions but can enter cells when the cell membrane is compromised. This study elucidates the diffusion mechanism of α-Syn in the pathogenesis of PD.

#### 6.1.5 Toxicity model of 6-hydroxydopa concentration gradient

The researchers cultured PC12 cells in a microfluidic channel and utilized the forward and backward movement of the fluid to establish a 6-OHDA concentration gradient within the channel. Subsequently, apoptosis was analyzed along the gradient. The results indicated that at higher concentrations, 6-OHDA primarily induced cell death through necrosis. Therefore, this concentration can serve as an effective *in vitro* model for PD by inducing the maximum level of apoptosis in PC12 cells (Seidi et al., 2011).

#### 6.1.6 Comprehensive analysis and mechanism discussion

In addition, as previously discussed, integrated computational simulations and *in vitro* experiments have validated the impact of chemical gradients (including inflammatory factors and metabolites) on cellular communication between nerve cells and the BBB. Research has demonstrated that variations in these gradients can substantially modulate transcellular signaling pathways, resulting in alterations in cytokine expression profiles and functional impairments (Voirin et al., 2020).

In conclusion, these findings underscore the critical role of microenvironment gradient disruption in neurodegenerative changes and α-syn pathological aggregation during the pathological progression of PD. Furthermore, they substantiate the feasibility of developing microenvironment chip models of PD from multiple perspectives, thereby providing a robust foundation for subsequent model validation and data analysis.

### 6.2 Strategies for model validation

In order to validate the functional performance of the chip model under gradient regulation, a range of experimental methods have been employed for model characterization: (1) TEER measurement: Transendothelial electrical resistance (TEER) measurement allows for real-time monitoring of the integrity and permeability changes in the BBB model. A decrease in the TEER value typically signifies damage to tight junction proteins and impairment of barrier function (Fernandes et al., 2016). (2) Fluorescence tracer analysis: Fluorescently labeled tracer molecules are utilized to detect substance transport across the barrier, enabling quantitative analysis of molecular transport influenced by gradient changes through real-time monitoring of fluorescence intensity and distribution (Frost et al., 2019; Noorani et al., 2021). (3) Molecular biomarker detection: Techniques such as real-time quantitative PCR, Western blot, or immunofluorescence are applied to assess expression changes in key signaling molecules (e.g., HIF-1α, claudin-5, occludin), thereby verifying the effects of gradient regulation at the molecular level.

Through these methods, we can not only evaluate the overall performance of the model at a macro level but also gain a deeper insight into the impact of gradient regulation on cell behavior at a micro level, thereby ensuring the reliability and validity of the model.

### 6.3 Data integration and multi-scale analysis

After validating the reliability of the model, the subsequent step involves analyzing experimental data to comprehensively reveal the impact of gradient adjustment on microflow control chips. Currently, the experimental data is integrated with computational simulations and multi-group datasets, enabling multi-scale and holistic analyses. At the cellular level, the experimental data regarding changes in cell behavior were analyzed to reflect the effects of gradient adjustment on cell function. At the systemic level, multiple datasets were consolidated, incorporating gene expression profiles and protein modifications, to elucidate the response mechanisms of cells to microenvironmental gradients. This approach ultimately achieves a comprehensive understanding of the influence of gradient adjustments.

#### 6.3.1 Computational fluid dynamics simulation

Computational fluid dynamics (CFD) technology can accurately model the fluid dynamics behavior within the chip, simulate gradient distribution, shear force fields, and solute dispersion. By constructing a chip model based on numerical calculations (such as finite element analysis), researchers can predict the distribution patterns of wall concentration and shear force under various operating parameters (flow rate, gradient amplitude, and geometric structure). This provides a theoretical foundation for experimental design and in-depth analysis of the gradient regulation mechanism (Shen et al., 2014; Noorani et al., 2021). Such multi-parameter numerical simulations not only aid in optimizing chip design but also elucidate the key variables and control points involved in the processes of gradient formation and transfer.

#### 6.3.2 Integrated analysis of multi-omics data

The integrated analysis of multi-omics data offers a comprehensive framework for elucidating the molecular mechanisms underlying cellular responses to microenvironmental gradient stimulation. By combining transcriptome data from RNA-seq with proteome data detected via mass spectrometry, a complete regulatory network of gradient signals on cell function can be revealed. Specifically, at the gene expression level, differential expression analysis enables the precise identification of dynamic patterns of key regulatory genes (e.g., NF-κB, HIF-1α, and components of the WNT pathway) under gradient conditions, thereby clarifying the hierarchical relationships and regulatory logic of signaling pathway activation. Furthermore, at the protein level, quantitative analysis of the expression abundance and modification states of tight-junction proteins, inflammatory factors, and metabolism-related proteins directly validates the immediate effects of chemical/physical gradients on cellular structural and functional remodeling. To establish the correlation between molecular mechanisms and the physical environment, we innovatively coupled CFD simulation data with multi-omics detection results to construct a cross-scale quantitative model. This model facilitates full-chain mechanism analysis, bridging fluid mechanics parameters to molecular event responses. This integrative analysis strategy, spanning from molecular characteristics to system behavior, not only provides novel insights into the mechanisms of microenvironmental gradient imbalance in the pathological progression of PD but also establishes a robust data foundation for the development of targeted intervention strategies by identifying critical driving factors (Shen et al., 2014).

## 7 Current challenges, limitations, and future prospects

### 7.1 Standardization of technology and modeling

In recent years, microfluidic chip technology has shown significant advancements in constructing BBB organoids and simulating complex *in vitro* microenvironmental gradients. However, critical challenges persist in achieving technical standardization and ensuring model reproducibility. Current microfluidic chip manufacturing processes exhibit substantial variability across research groups, particularly regarding substrate materials (such as PDMS, glass, thermoplastic polymers) and fabrication methodologies. These variations lead to inconsistencies in microstructural geometries, surface modifications, and channel designs, thereby undermining batch-to-batch reproducibility and inter-platform stability (Saorin et al., 2023). For example, differences in fluid dynamic parameters, gradient generation accuracy, and sensor integration protocols across chip designs directly compromise the comparability of experimental outcomes, such as drug permeability assays. Moreover, while real-time monitoring modules (e.g., in-line sensors, live-cell imaging systems) are continuously being refined, their long-term operational stability and durability require thorough validation. To address these issues, interdisciplinary collaboration is essential to prioritize standardized chip designs (e.g., 3D-printed modular chips) and open-access manufacturing protocols, particularly for resolving challenges related to functional unit interface standardization and long-term system reliability.

The long-term maintenance and dynamic monitoring of BBB organoids pose significant technical challenges. While current multicellular co-culture models effectively mimic key features of the *in vivo* microenvironment, they often fail to maintain functional stability over extended culture durations (ranging from weeks to months). Factors such as heterogeneous cellular proliferation, complex cell-matrix interactions, and temporal variations in biochemical gradients collectively lead to a gradual loss of endothelial cell polarity and impaired transmembrane transport, which can compromise the accuracy of high-throughput drug screening data (Cui and Cho, 2022; Dao et al., 2024). Importantly, integrating dynamic perfusion systems with mechanical stress simulations (e.g., pulsatile flow) represents a promising strategy. By finely tuning shear stress patterns and optimizing the temporal delivery of nutrients, these systems can sustain endothelial barrier integrity and metabolic activity. Nevertheless, achieving robust synchronization between biomechanical stimuli and biochemical microenvironmental cues remains a critical hurdle for improving model durability.

### 7.2 Physiological correlations and differences between *in vivo* and *in vitro* studies

While *in vitro* microfluidic BBB models enable precise control over biochemical gradients, mechanical stresses, and cellular interactions, they remain physiologically incomplete compared to *in vivo* systems. Key limitations include inadequate recapitulation of multicellular crosstalk (e.g., neuroimmune interactions), systemic metabolic coupling, and dynamic extracellular matrix remodeling. Current platforms often prioritize isolated parameter validation (e.g., single gradient effects) over multiscale integration, leading to fragmented interpretations of disease mechanisms. Bridging *in vitro* data with clinical relevance requires advanced analytical frameworks for cross-scale parameter correlation (molecular to organ-level) and incorporation of patient-derived cells or multi-organ interfaces. Emerging solutions leveraging machine learning and adaptive biosensor networks may enhance physiological fidelity, but achieving clinically predictive models demands systematic mimicry of self-regulating homeostasis and longitudinal pathophysiological progression.

### 7.3 Multi-technology integration and future development trends

In order to address the existing challenges, future research should focus on further exploration in technology integration and interdisciplinary collaboration, thereby facilitating the development of chip models toward high throughput, precision, and multi-system co-modeling.

First of all, using AI and big data technology, a large number of experimental data in the microfluidic chip can be mined and model optimized to achieve parameter optimization and high-throughput screening. For example, by analyzing different flow velocity, gradient amplitude and cell response data through machine learning algorithms, it is expected to establish a more accurate *in vitro* and *in vivo* association database and realize the construction of predictive models (Lashkaripour et al., 2021; McIntyre et al., 2022). This kind of data-driven analysis will provide strong support for chip design and optimization of experimental parameters. Secondly, in the future, in the field of chip manufacturing and functional expansion, emerging methods such as CRISPR gene editing technology, advanced sensor technology, and 3D printing are expected to further enhance the quality and functionality of model construction. By regulating key signaling pathways through CRISPR technology, integrating real-time monitoring sensors to precisely detect gradient changes, and reconstructing complex structures with 3D printing technology, these approaches can provide innovative ideas for improving chip design (de Almeida Monteiro Melo Ferraz et al., 2020; Zhou et al., 2020; Chen et al., 2022; Monia Kabandana et al., 2022). Additionally, the construction of cross-organ chips (such as brain-intestine and brain-liver combined models) will help reveal the interrelationship mechanisms of multi-system diseases and promote the integrated application of *in vivo* and *in vitro* models (Monia Kabandana et al., 2022).

Finally, it is recommended that standards be established through interdisciplinary collaboration. There is an urgent need to consolidate resources from multiple disciplines, including engineering, biology, clinical medicine, and computer science, to collaboratively develop a standardized framework for chip manufacturing, experimental procedures, and data analysis. This will effectively reduce the discrepancies between *in vivo* and *in vitro* studies and enhance the physiological relevance of the model. The trend toward interdisciplinary collaboration and multi-technology integration will offer advanced technical support and a robust theoretical foundation for exploring the mechanisms underlying neurodegenerative diseases such as Parkinson’s disease and other complex conditions.
